# Seventh International Congress of Veterinary Surgeons

**Published:** 1899-05

**Authors:** 


Secretary.

### SEVENTH INTERNATIONAL CONGRESS OF VETERINARY SURGEONS.

The Seventh International Congress of Veterinary Surgeons will take place from August 7 to 12, 1899, in Baden-Baden. The persons that, according to the statutes, will be admitted regular members of the Congress are the representatives of governments, town administrations, universities, veterinarian and agricultural colleges and institutes for the care of public health, as well as those deputies of veterinary and agricultural societies, veterinary surgeons and surgeons that have paid the personal fee of twelve marks, or twelve shillings, or fifteen francs at the branch of the Rheinische Creditbank in Baden-Baden. This fee gives a right to post-free receipt of all the publications of the sessions and reports of the Congress, including the general report.

For the Committee of Direction, Dr. Lydtin, the President; Dr. Casper, the Secretary-General.

The programme is as follows:

*Sunday, August* 6, 1899, at 8 P.M. Reception of the guests and members of the Congress in the hall of the Conversation House restaurant.

*First Principal Session of the Congress, Monday, August* 7, 1899, *at* 9 A.M. Greeting of the invited guests; addresses of the imperial, grand ducal government and town authorities; election of the honorary presidents; report of the chairmen of the committee of direction; determining of the regulations; appointment of honorary members; election of the bureau.

Discusssion of "Preventive Measures against the Spread of Epizoötics in Consequence of International Cattle-trade." Reporters: Mr. Brändle, Chief Veterinary Surgeon of the Canton of St. Gall, Switzerland; Mr. Cope, Chief Veterinary Officer of the Board of Agriculture in London; Dr. Hutyra, Director and Professor of the Royal Academy of Veterinary Medicine, Budapest; M. Camille Leblanc, Member of the Academy of Medicine, Paris; Dr. Lothes, Chief Veterinary Surgeon at Cologne.

*Second Principal Session, Tuesday, August* 8, 1899, *at* 9 A.M. "The Prevention of Foot-and-mouth Disease." Reporters: Mr. Cagny, Member of the Central Society of Veterinary Medicine, Senlis, Oise; Mr. Cope, Chief of the Veterinary Service at the Board of Agriculture, London; Dr. Dammann, Privy Councillor, Director and Professor of the Veterinary High

School in Hanover; Mr. Furtuna, Chief Veterinary Officer of the Board of Agriculture in Bucharest; Mr. Hafner, Councillor of the Government, Karlsruhe; Mr. Hess, Professor of the School of Veterinary Medicine, Berne; Mr. Lindqvist, former Director and Professor of the Institute of Veterinary Medicine, Stockholm.

*Third Principal Session, Wednesday, August* 9, 1899, *at* 9 A.M. 1. "The Newest Suggestions for an Effectual Meat-inspection." Reporters: Dr. Edelmann, Head Inspector of Slaughter-houses, Dresden; Mr. Gustave Kjerrulf, Superior Veterinary Surgeon, Stockholm; Mr. Postolka, Superior Veterinary Surgeon, Vienna.

2. "The Announcement of the Final Result of the Endeavors to Fix a Uniform Anatomical Nomenclature in Veterinary Medicine." Reporters: Dr. Arloing, Director and Professor of the National College of Veterinary Medicine, Lyons; Dr. Ellenberger, Chief Medical Councillor, Professor of the College of Veterinary Medicine, Dresden; Mr. Martin, Professor of the School of Veterinary Medicine, Zurich; Dr. Struska, Professor of the High College of Veterinary Medicine, Vienna; Dr. Sussdorf, Professor of the Veterinary High School, Stuttgart.

*Fourth Principal Session, Thursday, August* 10, 1899, *at* 8 A.M. 1. "The Prevention of Tuberculosis Among Domestic Animals." Reporters: Dr. Bang, Professor of the High School of Veterinary Medicine, Copenhagen; Mr. Regnér, Veterinary Surgeon at the Board of Agriculture at Stockholm; Dr. O. Malm, Chief of the Veterinarian Office of Norway; Dr. Siedamgrotzky, Privy Councillor, Professor of the High School of Veterinary Medicine, Dresden; Dr. Stubbé, Veterinary Inspector of the Board of Agriculture, Brussels.

2. "The Use of Flesh and Milk of Tuberculous Animals." Reporters: Mr. Butel, Member of the Central Society of Veterinary Medicine, Meaux; Mr. de Jong, Veterinarian of the Netherlands, Leyden; Dr. Ostertag, Professor of the School of Veterinary Medicine, Berlin.

*Fifth Principal Session, Friday, August* 11, 1899, *at* 9 A.M. "The Prevention of Swine Epidemics." Reporters: Dr. Leclainche, Professor of the National School of Veterinary Medicine, Toulouse; Dr. Lorenz, Superior Medical Councillor, Darmstadt; Dr. Perroncito, Professor of the Superior Institute of Veterinary Medicine, Turin; Dr. Preusse, Chief Veterinary Surgeon, Danzig.

*Sixth Principal Session, Saturday, August* 12, 1899, *at* 9 A.M. 1. "The Extension of Veterinary Instruction, Especially by the Establishment of Institutes for Making Experiments in Epizoötic Diseases and of Chairs of Comparative Medicine in Veterinary High Schools." Reporters: Mr. Degive, Director and Professor of the Royal High School of Veterinary Medicine, Cureghem, Brussels; Dr. Kitt, Professor of the High School of Veterinary Medicine, Munich; Dr. Malkmus, Professor of the High School of Veterinary Medicine, Hanover; Dr. Nocard, Professor of the National High School of Veterinary Medicine in Alfort, Member of the Academy of Medicine; Dr. Nogueira, Professor of the School of Veterinary Medicine, Lisbon; Dr. Schutz, Privy Councillor, Professor of the High School of Veterinary Medicine, Berlin.

2. "Veterinary Officials." Reporter: Dr. Lydtin, Baden-Baden.

Determination of the time and place of the Eighth Congress; election of a commission for auditing the accounts; conclusion of the Congress; general banquet.

